# Examination of the relationship between D-amino acid profiles and cognitive function in individuals with mild cognitive impairment: a machine learning approach

**DOI:** 10.1093/ijnp/pyaf016

**Published:** 2025-03-15

**Authors:** Sou Sugiki, Shigeki Tsuchiya, Ren Kimura, Shun Katada, Koichi Misawa, Hisashi Tsujimura, Masanobu Hibi

**Affiliations:** Human Health Care Products Research, Kao Corporation, Tokyo, Japan; Analytical Science Research, Kao Corporation, Tokyo, Japan; Analytical Science Research, Kao Corporation, Tokyo, Japan; Human Health Care Products Research, Kao Corporation, Tokyo, Japan; Human Health Care Products Research, Kao Corporation, Tokyo, Japan; Analytical Science Research, Kao Corporation, Tokyo, Japan; Human Health Care Products Research, Kao Corporation, Tokyo, Japan

**Keywords:** dementia, mild cognitive impairment, D-amino acids, peripheral blood, machine learning, nonlinear models, early detection, screening tool

## Abstract

**Background:**

The global prevalence of dementia is significantly increasing. Early detection and prevention strategies, particularly for mild cognitive impairment (MCI), are crucial but currently hindered by the lack of established biomarkers. Here, we aimed to develop a high-precision screening method for MCI by combining D-amino acid profiles from peripheral blood samples with noninvasive subject information using nonlinear machine learning (ML) algorithms.

**Methods:**

A cross-sectional study was conducted with 200 participants aged 50–89 years, classified into cognitively normal and MCI-suspected groups based on Mini-Mental State Examination scores. High-throughput techniques were used to analyze the D-amino acid profiles, specifically D-alanine (%) and D-proline (%), in peripheral blood. Correlation analysis was performed between D-amino acid levels in venous and fingertip blood. The predictive performance of various ML models, including Logistic Regression, Random Forest, kernel Support Vector Machine (SVM), and Artificial Neural Network (ANN), was compared.

**Results:**

Nonlinear models (kernel SVM and ANN) that combined D-amino acid profiles with subject information achieved the highest area under the curve values of 0.78 and 0.79, respectively, demonstrating that the combination of D-amino acid profiles and noninvasive subject information is effective in detecting MCI.

**Conclusions:**

Combining D-amino acid profiles with noninvasive subject information using nonlinear ML models, particularly kernel SVM and ANN, shows promise as a high-precision screening tool for MCI. This approach could serve as a cost-effective preliminary screening method before more invasive and expensive diagnostic tests and significantly contribute to the early detection and development of intervention strategies for dementia.

**Significance Statement**

Dementia affects millions worldwide, significantly impacting individuals and caregivers. Early detection of mild cognitive impairment (MCI) can delay or prevent progression to dementia, but current methods are invasive and costly. This study combines D-amino acid profiles from fingertip blood samples with noninvasive subject information using advanced machine learning models to accurately detect MCI. Our approach demonstrated promising accuracy, suggesting it could serve as a cost-effective, noninvasive screening tool for early dementia risk, enhancing timely intervention and potentially reducing the global dementia burden.

## INTRODUCTION

Dementia results from neurodegenerative disorders of the brain and is characterized by the progressive deterioration of memory, judgment, and language functions. According to the World Health Organization,^1^ the number of people with dementia worldwide was estimated at 50 million in 2019 and is expected to increase to 82 million by 2030 and 152 million by 2050. Dementia is a serious problem that not only greatly reduces the quality of life for the individual but also places a tremendous burden on caregivers and their families; solving this problem is a major goal in medicine, nursing care, and society.^2^ Forms of dementia include Alzheimer’s disease (AD), vascular dementia, frontotemporal dementia, Lewy body dementia, and others. The detailed causes of onset are not yet fully understood, and the discovery of effective treatments is an urgent issue for the aging population worldwide. Mild cognitive impairment (MCI), in which cognitive function is declining but there is no significant impact on daily life, precedes dementia. Among individuals diagnosed with MCI, 10% develop dementia within a year.^3^ Through improvements in diet and lifestyle, however, 16%–41% of individuals diagnosed with MCI can return to a state of healthy aging.^4^ Therefore, practical strategies for dementia prevention include the earliest possible detection of the risk of dementia and the improvement of related lifestyle factors.

Diagnosing dementia requires extensive clinical testing, including brain imaging, cerebrospinal fluid (CSF) measurements of amyloid b (Ab) and tau protein, cognitive function evaluation, and behavioral assessment.^5,6^ These tests are highly invasive and time-consuming to administer, underscoring the need for highly sensitive and specific alternative biomarkers to enable earlier and easier detection. In recent years, researchers have intensified efforts toward identifying blood biomarkers for the early detection of dementia. As biomarkers in venous blood that reflect the accumulation of Ab in the brain, several methods have been reported, including an index combining the concentrations of 176 molecules measured in plasma,^7^ the plasma phosphorylated tau concentration,^8^ the plasma Ab 42/ Ab 40 ratio,^9-11^ and methods combining protein concentrations related to the clearance of serum Ab 40 and Ab.^12^ Although these blood biomarkers hold promise as future testing methods, effective methods of monitoring cognitive decline at an early time point and in a less burdensome manner have yet to be established.

D-amino acids, which are present in minimal amounts (<1%) in the blood and function as components of proteins and biological constituents, are associated with various diseases and aging and have attracted attention as potential biomarkers.^13,14^ Kimura et al.,^15^ focusing on blood D-amino acids as biomarkers for MCI detection, developed a high-throughput technology to analyze D-amino acids from small amounts of venous serum. The results of their comprehensive analysis of D-amino acids from blood samples of 305 women in a cross-sectional cohort study suggested that the enantiomer ratio might be useful as an evaluation index of cognitive function.^15^ Hashimoto et al.^16^ and Piubelli et al.^17^ reported decreases in D-serine (D-Ser) levels and the D-Ser/total Ser (D/T-Ser) ratio in AD patients compared with healthy individuals. These findings, however, have not been confirmed in comparative studies of D-amino acid profiles in CSF from patients with preclinical AD, MCI, or dementia. Furthermore, no correlation has been observed between serum and CSF D-Ser levels and scores on the Mini-Mental State Examination (MMSE) or Clinical Dementia Rating Scale.^18^

Thus, while reports on changes in blood amino acid profiles, particularly D-amino acid profiles, in individuals with dementia or MCI are increasing, the results are inconsistent and still under discussion. Furthermore, the considerable overlap between the enantiomeric ratios of D-amino acids in healthy and individuals with MCI makes classification based on cutoff values difficult, even for parameters that are significantly different. In addition, to accurately detect cognitive decline in a simple and less burdensome way for individuals, the detection accuracy of capillary blood sampling through fingertip puncture, in addition to venous blood sampling, should be evaluated.

This cross-sectional study was conducted to establish a highly accurate MCI differentiation method using D-amino acid profiles from capillary blood sampling with fingertip puncture in men and women classified based on MMSE scores as healthy or MCI-equivalent. The primary endpoint was the relationship between the D-amino acid enantiomer ratios and cognitive function classification by MMSE. A secondary aim of the study was to develop a nonlinear machine learning (ML) algorithm using D-amino acid enantiomer ratios and noninvasive participant data.

## MATERIALS AND METHODS

### Participants and Research Ethics

Participants were recruited by 3H Medi Solution Inc. through a website between September 2022 and December 2022. The study was conducted as a cross-sectional observational study at LSI Sapporo Clinic and Yamazaki Otolaryngology Clinic. The inclusion criteria were as follows: (1) men and women aged 50 to 89 years and (2) individuals who could fully understand the purpose and content of the study and lead an independent life. The exclusion criteria were as follows: (1) individuals diagnosed with dementia or taking medication to treat dementia; (2) individuals diagnosed with depression or taking medication for depression; (3) individuals who have undergone treatment, hospitalization, or surgery for head injuries such as stroke or subarachnoid hemorrhage; (4) individuals with malignant tumors, diabetes, liver disease, kidney disease, cardiovascular disease, respiratory disorders, endocrine disorders, metabolic disorders, consciousness disorders, or other serious diseases; (5) individuals with a history of neurodegenerative diseases, cardiovascular diseases, or thyroid disorders; 6) individuals infected with syphilis, HIV, hepatitis C virus, hepatitis B virus, or COVID-19; (7) individuals taking medication that may affect cognitive function (antipsychotics, anxiolytics, antidepressants, etc.); (8) heavy alcohol drinkers (an average of 60 g or more of pure alcohol per day); (9) other individuals deemed inappropriate for participation in this study by the principal investigator.

Evaluation and screening of cognitive function were conducted at LSI Sapporo Clinic in September 2022, and blood sampling was conducted at Yamazaki Otolaryngology Clinic from September 2022 to December 2022. This study was conducted in accordance with the tenets of the Declaration of Helsinki, and the study protocol was reviewed and approved by the Ethics Committee of Kao Corporation (approved on August 2, 2022, approval number K0117-2206) and the Ethics Committee of Yamazaki Otolaryngology Clinic (approved on July 5, 2022, approval number 2206K). The study was registered in the University Hospital Medical Information Network Clinical Trials Registry (https://www.umin.ac.jp/, UMIN000054494) before participant recruitment. All participants received an explanatory document and provided written informed consent before participating in the study. The required sample size was calculated using data from previous studies and unpublished preliminary results, with the D/T-proline (Pro) ratio (%) ±  SD being 0.50 ± 0.34 for individuals with MMSE scores ≤ 26 and 0.35 ± 0.20 for those with MMSE scores ≥ 27. Based on these data, a sample size of 170 participants was calculated (1-b = 80%, a = 5%), and allowing for a dropout rate of 10%; the target sample size was set at 200.

### Participant Information and Cognitive Function Evaluation

Trained physicians or research coordinators collected information on participants’ sex, age, years of education, number of cohabitants and family composition, occupation, medical history, current diseases under treatment, medication, smoking habits, drinking habits, and caregiving certification for those aged ≥75 years. Height was measured using a stadiometer, and weight was measured using a digital bioimpedance scale (InBody 770K, InBody Corp.) by trained research coordinators and recorded to the nearest decimal point (kg). Body mass index (BMI, kg/m^2^) was calculated by dividing the weight (kg) by the square of the height (m). Cognitive function was evaluated by a certified psychologist at LSI Sapporo Clinic based on neuropsychological assessments. General cognitive function was assessed using the MMSE, administered as a series of questions divided into 11 categories (time orientation, place orientation, memory, attention, recall, naming, repetition, comprehension, reading, writing, and drawing). Based on previous reports,^15^ individuals scoring 27–30 points were classified into the cognitively normal group (CN) group, and those scoring 22–26 points were classified into the suspected MCI (sus-MCI) group.^19^

### D-Amino Acid Analysis

Participants underwent venous and fingertip blood sampling after overnight fasting. Venous blood was collected from the forearm vein into tubes containing the anticoagulant ethylenediaminetetraacetic acid disodium salt (EDTA-2Na; Terumo Corporation), and fingertip blood was collected into micro blood sampling capillaries containing the anticoagulant EDTA-2Na (MBS Corporation). Both blood samples were mixed by inversion after collection, and plasma samples were obtained by centrifugation of the collected blood samples (1500 × *g*, 4 °C, 10 min). Blood samples were analyzed by Kao Corporation following the method of Kimura et al.^15^ with DL-amino acid quantification and enantiomer ratios calculated for alanine (Ala), Pro, and Ser. The enantiomeric proportion (D-amino acid [%]) was calculated as a percentage of the D-form to the total (D + L) concentration based on the peak area.

### Preprocessing of ML Data

A ML model was constructed using the obtained data, including D-amino acid (%), sex, age, years of education, number of cohabitants, family composition, and BMI. To enable appropriate learning by the ML model, data were preprocessed by type as follows. Mini-Mental State Examination scores were used as explanatory variables for the classification task of the sus-MCI (MMSE ≤ 26) and CN (MMSE ≥ 27) groups, with the sus-MCI group labeled as 1 and the CN group labeled as 0, creating the training data. D-amino acid (%) values were standardized by item to allow for equitable learning with participants’ background information using the following formula, where μ represents the mean of the original data and σ represents the SD.


$$\begin{array}{l} x_{std}^{i}=\frac{x^{i}-\mu }{\sigma }\; \end{array}$$

Participant background information was preprocessed according to the variable type as follows. Age, years of education, BMI, and number of cohabitants were standardized in the same way as D-amino acid (%). Family composition was represented in binary form, indicating the presence or absence of a spouse, siblings, and children. Sex was coded as male (0) or female (1).

### Machine Learning

An ML model was applied to classify the labeled sus-MCI and CN groups. In this study, we compared a linear separable model, the logistic regression model, and nonlinear separable models, including the random forest (RF) model,^20^ the support vector machine using a kernel method (kernel SVM),^21^ and an artificial neural network model (ANN).^22^ The SVM with a kernel employed a Gaussian kernel (RBF kernel). In the ANN, preprocessed data were fed into the input layer, followed by an intermediate layer with 100 nodes, both using the ReLU function activation function. The final output layer, containing a single node, utilized the Sigmoid activation function. The ANN model was optimized using stochastic gradient descent to form the final model.

Given that MMSE scores of ≤27 are often used to classify MCI,^23^ we investigated the model’s performance when sus-MCI was defined as MMSE ≤ 27 for post hoc analysis to analyze the robustness of the model. As the boundary between CN and sus-MCI is difficult to determine using the MMSE and label accuracy is limited, we also assessed model performance after excluding data with MMSE scores of 26 and 27, which fall within this boundary region. The entire series of steps for model construction, training, and robustness analysis was implemented using Python 3.10.10.

### Statistical Analysis

Unless otherwise specified, all clinical characteristics are expressed as mean ± SE. Using Spearman’s rank correlation test, a correlation analysis of the concentrations of D/T-Ser ratio (D-Ser [%]), D/T-Pro ratio (D-Pro [%]), and D/T-Ala ratio (D-Ala [%]) in venous and fingertip blood was performed. The medians were compared between groups using the Mann-Whitney U test. When covariates were suspected, an analysis of covariance (ANCOVA) was performed. The accuracy, area under the curve (AUC), sensitivity, specificity, positive predictive value (PPV), and negative predictive value (NPV) of D-amino acid (%) were verified using the optimal threshold maximizing Youden’s J statistic obtained from the receiver operating characteristic (ROC) curve. Additionally, predictions from various ML models were used to calculate accuracy, AUC, sensitivity, specificity, PPV, and NPV to evaluate model performance. Leave-one-out cross-validation (LOOCV) was implemented using Python 3.10.10. A *P*-value of less than.05 was considered statistically significant. All statistical analyses were performed using R 4.3.2 software (R Foundation).

## RESULTS

### Participants

**Figure 1.**
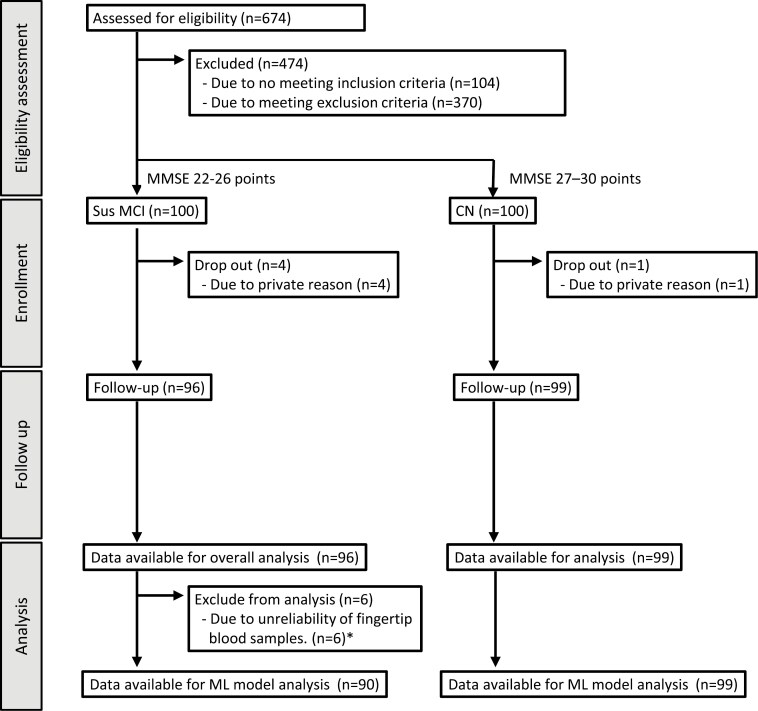
Clinical trial flow. *In the comparison of the correlation between venous plasma and fingertip whole blood for D-Pro (%) or D-Ala (%), outliers are defined as those with standardized residuals exceeding 2.0.

The CN group included 50 men and 50 women, and the sus-MCI group included 50 men and 50 women. Five participants (1 woman from the CN group, 1 man, and 3 women from the sus-MCI group) dropped out of the study, resulting in final participant numbers of 50 men in the CN group, 49 men in the sus-MCI group, 49 women in the CN group, and 47 women in the sus-MCI group (Figure 1). The average MMSE scores for the groups were as follows: CN-Men, 28.8 (SD 0.8); sus-MCI-Men, 24.2 (SD 0.8); CN-Women, 28.6 (SD 0.8); and sus-MCI-Women, 23.6 (SD 2.1). Mean participant age in the respective groups was CN-Men, 72.8 years (SD 8.0); sus-MCI-Men, 75.4 years (SD 8.0): CN-Women, 74.5 years (SD 5.3); and sus-MCI-Women, 80.6 years (SD 9.1). The participants’ physical information, cognitive function, and venous plasma data obtained in the study are shown in Table 1.

**Table 1.** Characteristics of older individuals with cognitively normal (CN) and those with suspected mild cognitive impairment (sus-MCI).

	CN, *M(SD) n *= 100	sus-MCI, *M(SD) n* = 97	*P* value^a^
Age (years)	73.65 (6.81)	77.43 (8.99)	.002
BMI (kg/m^2^)	23.24 (3.30)	23.09 (4.09)	.597
Education^b^ (years)	12.77 (2.25)	10.9 (2.39)	<.001
No. of people living together	1.03 (0.91)	0.74 (0.98)	.005
WMS-R LM II	13.57 (8.79)	5.93 (6.64)	<.001
Pulse	71.1 (10.91)	70.19 (10.65)	.468
ALT (U/L)	20.41 (13.40)	16.90 (7.80)	.101
CK (U/L)	112.85 (65.73)	94.16 (65.41)	.005
γ-GTP (U/L)	35.92 (29.64)	25.19 (18.63)	.002
ALP (U/L)	76.87 (23.34)	74.52 (24.69)	.677
BUN (mg/dL)	16.37 (4.85)	15.51 (6.9)	.035
T-CHO (mg/dL)	198.49 (38.84)	219.07 (42.01)	<.001
HDL-CHO (mg/dL)	59.71 (16.46)	69.03 (17.86)	<.001
LDL-CHO (mg/dL)	113.02 (32.77)	124.92 (34.73)	.014
TG (mg/dL)	112.5 (66.63)	101.12 (43.19)	.643
BS (mg/dL)	97.66 (18.14)	94.83 (15.15)	.204
HbA1c (%)	5.7 (0.63)	5.63 (0.61)	.465

^a^ *P* value; Mann-Whitney U test between the CN group and the sus-MCI group.

^b^Education; years of schooling.

Abbreviations: ALP, alkaline phosphatase; ALT, alanine aminotransferase; BUN, blood urea nitrogen; BS, blood sugar; CHO, cholesterol; CK, creatine kinase; γ-GTP, γ-glutamyltransferase; HbA1c, hemoglobin A1c; HDL, high-density lipoprotein; LDL, low-density lipoprotein; TG, triglyceride; WMS-R LM II, Wechsler Memory Scale-Revised Logical Memory II.

### Correlation Analysis of D/T-Amino Acid Levels in Venous Plasma and Whole Fingertip Blood

**Figure 2.**
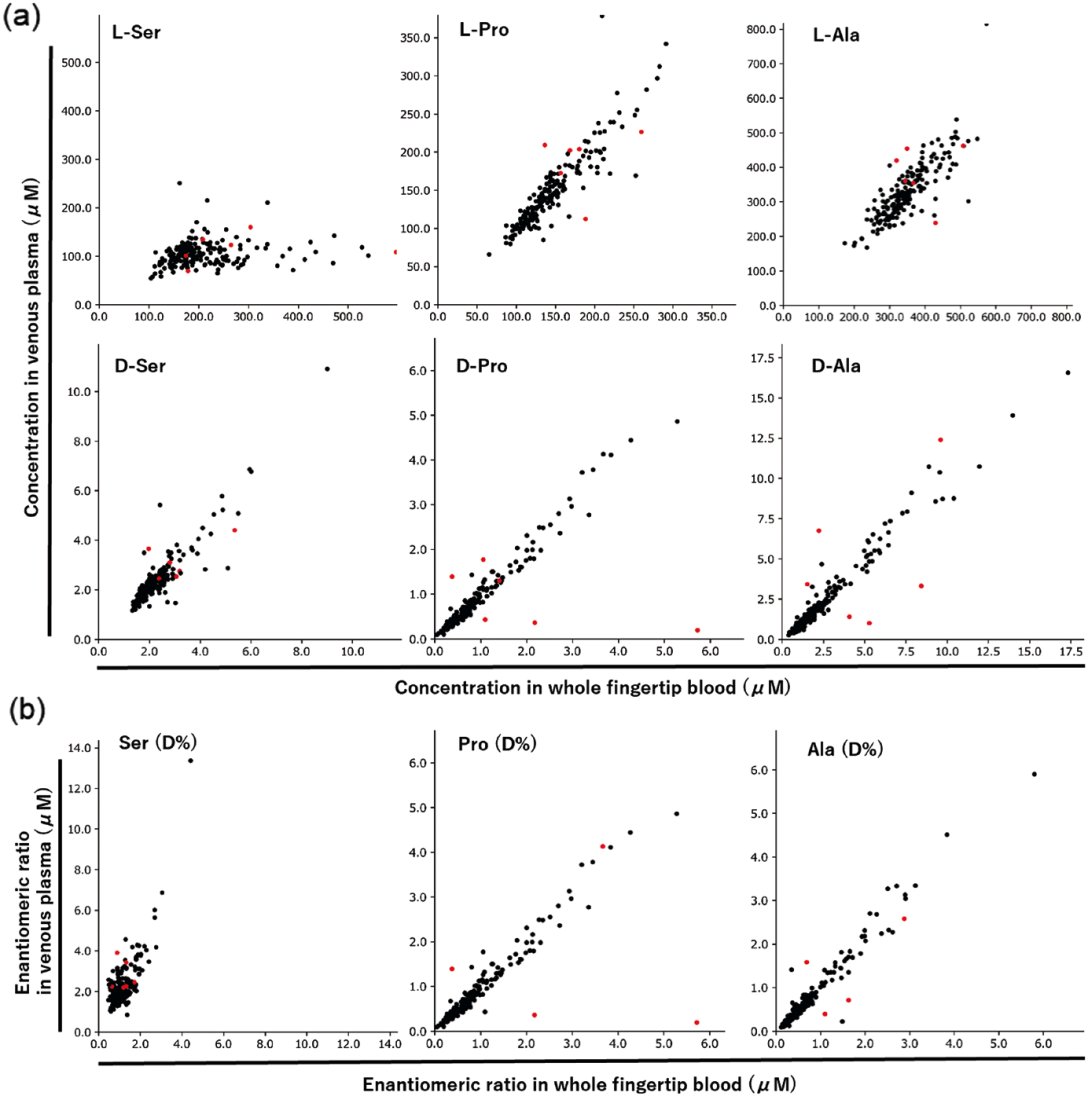
(A) Correlation comparison of amino acid concentrations in venous and fingertip blood. Red dots indicate data points with outliers. (B) Correlation comparison of amino acid concentrations and D-amino acid (%) in venous and fingertip blood, excluding outliers.

The concentrations of each amino acid and each D-amino acid (%) in the venous and fingertip blood of all participants, including the CN and sus-MCI groups, were compared (Figure 2). The venous and fingertip blood samples showed high correlations in D-Ser (%), L-Pro levels (L-Pro), D-Pro (%), L-Ala levels (L-Ala), and D-Ala (%) values, with correlation coefficients of *r* = 0.90, *r *= 0.90, *r* = 0.87, *r* = 0.82, and *r *= 0.97, respectively. On the other hand, L-Ser levels showed a low correlation between venous and fingertip blood samples (*r* = 0.18). Thus, the correlations of the enantiomeric ratio of Pro and Ala were high between venous and fingertip blood, but that of Ser was low (*r* = 0.76). Higher correlation coefficients were observed when points with absolute values of standardized residuals of D-Pro (%) or D-Ala (%) > 2.0 were removed as outliers (6 subjects) (D-Pro [%], *r* = 0.98; D-Ala [%], *r *= 0.99; D-Ser [%], *r* = 0.78). For subsequent analyses, we used D-Ala (%) and D-Pro (%), excluding the low correlation D-Ser (%) and 6 outlier cases.

### D/T-Amino Acid Analysis in Whole Fingertip Blood

**Figure 3.**
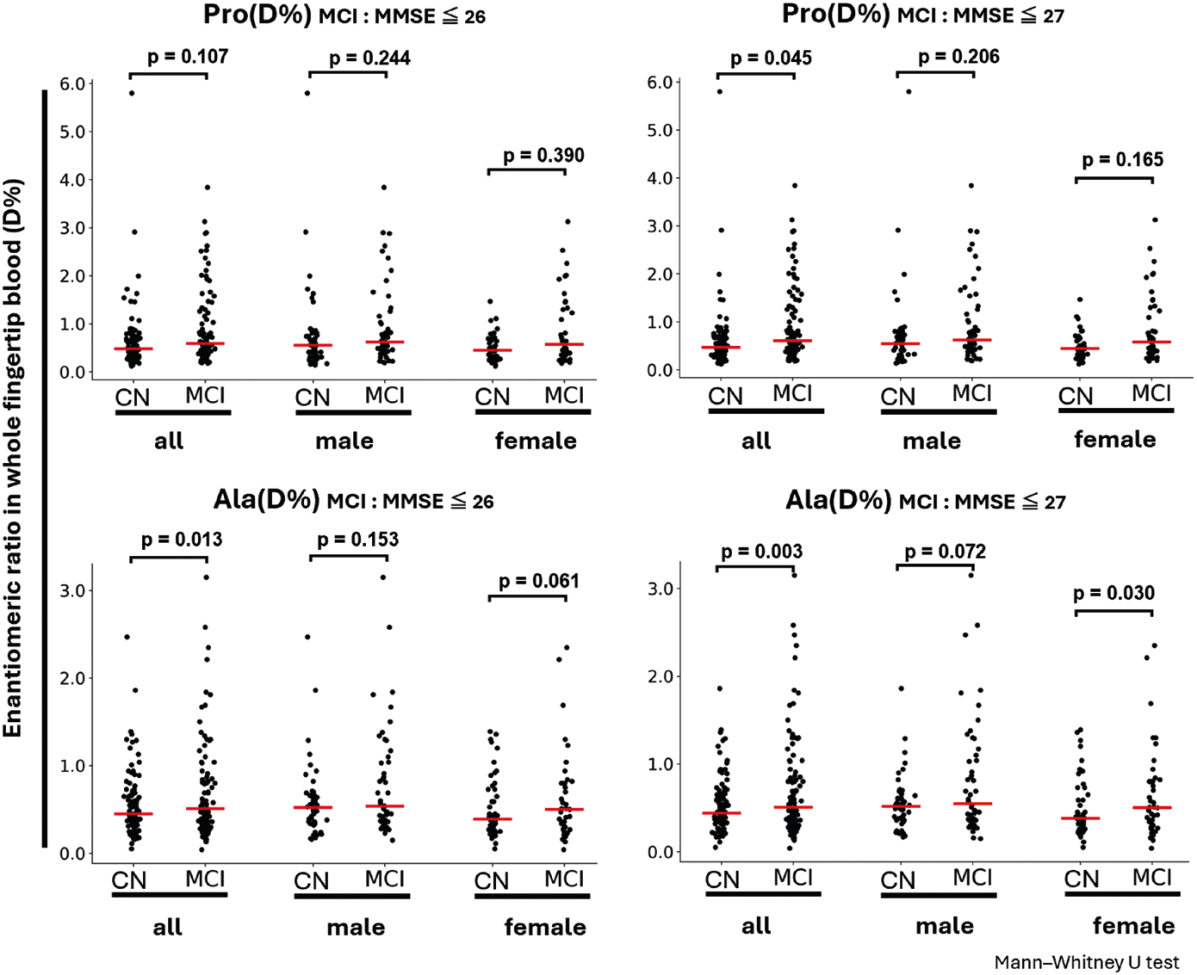
Scatterplots showing group differences in the concentration of D-Pro (%) and D-Ala (%) in whole fingertip blood. Black horizontal lines denote the median value. Differences in each concentration between the cognitively normal (CN) group and the suspected MCI group (MCI) by sex were explored using the Mann-Whitney U test. MCI, mild cognitive impairment.

The Jitter plots of D-Ala (%) and D-Pro (%) for the entire CN group and sus-MCI group, men, and women, with the MCI threshold set to MMSE ≤ 26, and MMSE ≤ 27, which is currently used in many studies, are shown in Figure 3. The Mann-Whitney U test showed a significant increase in overall D-Ala (%) in the sus-MCI group compared to the CN group (*P* = .013), and a trend toward an increase in D-Ala (%) in women (*P* = .061) with the MCI threshold set to MMSE ≤ 26. With the widely used threshold of MMSE ≤ 27, a significant increase in D-Ala (%) was observed in the overall and female sus-MCI groups compared to the CN group (*P* = .003 and *P* = .030, respectively), and a trend toward an increase in D-Ala (%) was observed in men (*P* = .072). When a threshold of MMSE ≤ 27 was used, a significant increase in D-Pro (%) was observed in the overall sus-MCI group compared to the CN group (*P* = .045). No differences were observed in the enantiomeric ratio of D-Ser (Figure S1). Regarding the results with significant differences and trends between groups in the comparison of D-Ala (%) and D-Pro (%), ANCOVA was conducted to adjust for age, years of education, and the number of people living together as covariates, which were correlated with MMSE. In the females, a significant increase in D-Ala (%) was observed with the MCI threshold set to MMSE ≤ 26 (*P* = .039). An increasing tendency was observed in D-Ala (%) in the sus-MCI group compared to the CN group in the overall comparison when a threshold of MMSE ≤ 26 was used (*P* = .098). In female, an increasing tendency for D-Pro (%) was observed in the sus-MCI group compared to the CN group (*P* = .061). Additionally, the rank correlation coefficients between MMSE scores and D-amino acid (%) and subject information used for ML are shown in Table 2. Significant positive correlations were observed for age, years of education, and number of cohabitants, and significant negative correlations were observed for D-Ala (%) and D-Pro (%). A positive correlation trend was observed for BMI.

**Table 2.** Rank correlation coefficients between parameters used in machine learning and MMSE* scores.

	Correlation coefficient	*P*-value
Age (years)	0.421	<.001
BMI (kg/m^2^)*	0.147	.044
Education(years)	0.421	<.001
No. of cohabitants	0.308	<.001
D-Ala%(%)	−0.266	<.001
D-Pro%(%)	−0.215	<.001

*Abbreviations: BMI, body mass index; MMSE, Mini-Mental State Examination.

### Classification by Enantiomer Ratio and ML Model Prediction Accuracy

Using ROC curves, D-amino acid (%) values, and noninvasively obtainable participant background information (sex, age, BMI, years of education, number of cohabitants, and family composition) as explanatory variables, we evaluated the LOOCV performance of various models predicting sus-MCI as classified by MMSE scores. The results are shown in Table 3. Compared to classification based solely on D-amino acid (%) thresholds, ML using D-amino acid (%) and background information demonstrated higher performance. Among the ML algorithms, nonlinear separable models (RF, kernel SVM, ANN) outperformed the linear separable model. The kernel SVM and ANN models achieved the highest performance, with AUCs of 0.78 and 0.79, respectively. Given its fast processing speed and relatively easy model interpretation, we selected the kernel SVM as the best model for further analysis.

**Table 3.** Comparison of performance based on discrimination of the cognitively normal group and suspected mild cognitive impairment group.^a^

Algorithm	Accuracy	AUC	Sensitivity	Specificity	PPV	NPV
Thresholds^b^ (D-Ala% = 1.0)	0.619	0.605	0.311	0.899	0.737	0.589
Thresholds^b^ (D-Pro% = 0.75)	0.598	0.568	0.367	0.808	0.635	0.584
Logistic regression^c^	0.651	0.710	0.589	0.707	0.646	0.654
Random forest^c^	0.672	0.726	0.578	0.758	0.684	0.664
Kernel SVM^c^	0.762	0.782	0.711	0.808	0.771	0.755
ANN^c^	0.767	0.790	0.722	0.808	0.774	0.762

^a^Performance based on the discrimination method was compared using Accuracy (the proportion of true results among the total cases), AUC (area under the curve, a measure of the ability of a classifier to distinguish between classes), Sensitivity (true positive rate), Specificity (true negative rate), PPV (positive predictive value; proportion of positive results that are true positives), and NPV (negative predictive value; proportion of negative results that are true negatives).

^b^Thresholds for D-Ala% and D-Pro% were set to maximize Accuracy.

^c^Performance comparison of each machine learning model using LOOCV (leave-one-out cross-validation).

Abbreviations: ANN, artificial neural network; SVM, support vector machine.

### Contribution of D-Amino Acid (%) to the Model

**Figure 4.**
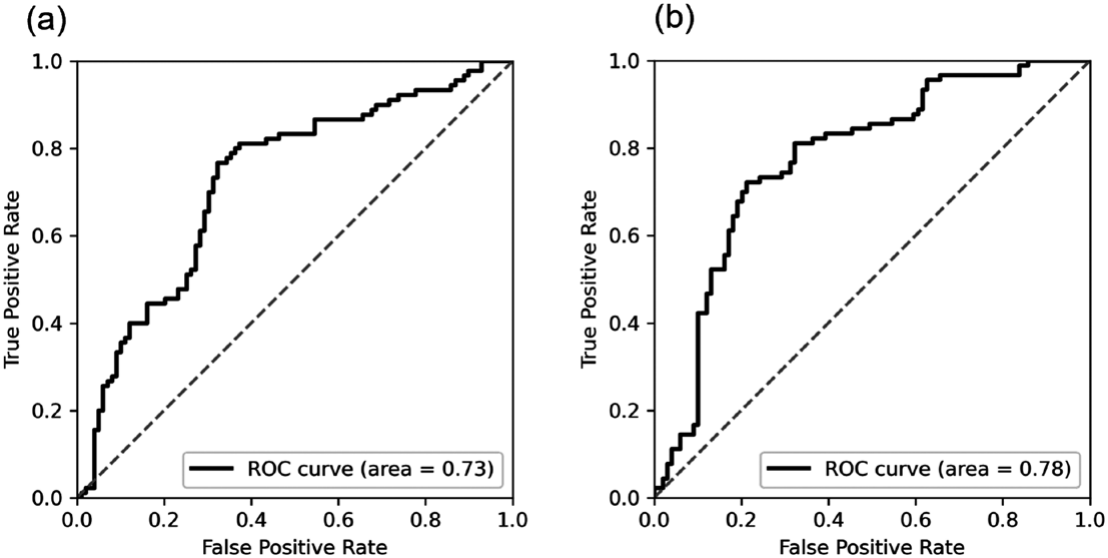
(A) The ROC curve when a kernel SVM model was constructed using relatively easily obtainable participant background information (sex, age, BMI, education years, number of people living together, and family composition), and LOOCV was performed. (B) The ROC curve when a kernel SVM model was constructed using the information from (A) and enantiomeric ratio information (D-Ala [%], D-Pro [%]), and LOOCV was performed. BMI, body mass index; LOOCV, leave-one-out cross-validation; ROC, receiver operating characteristic; SVM, support vector machine.

To investigate whether D-amino acid (%) contributed to the improvement in model performance, we constructed a kernel SVM model using only participant background information (sex, age, BMI, years of education, number of cohabitants, and family composition) and compared it to the combined model using both enantiomer ratios and background information. The model using only background information had an accuracy of 0.682, AUC of 0.725, sensitivity of 0.656, specificity of 0.707, PPV of 0.670, and NPV of 0.693, all of which were lower than the combined model. Figure 4 shows the ROC curves for (1) the model using only background information and (2) the combined model. The combined model demonstrated a steeper rise in the ROC curve, suggesting the potential to enhance sensitivity while maintaining high accuracy depending on the threshold.

### Robustness Analysis Based on MMSE Criteria

**Figure 5.**
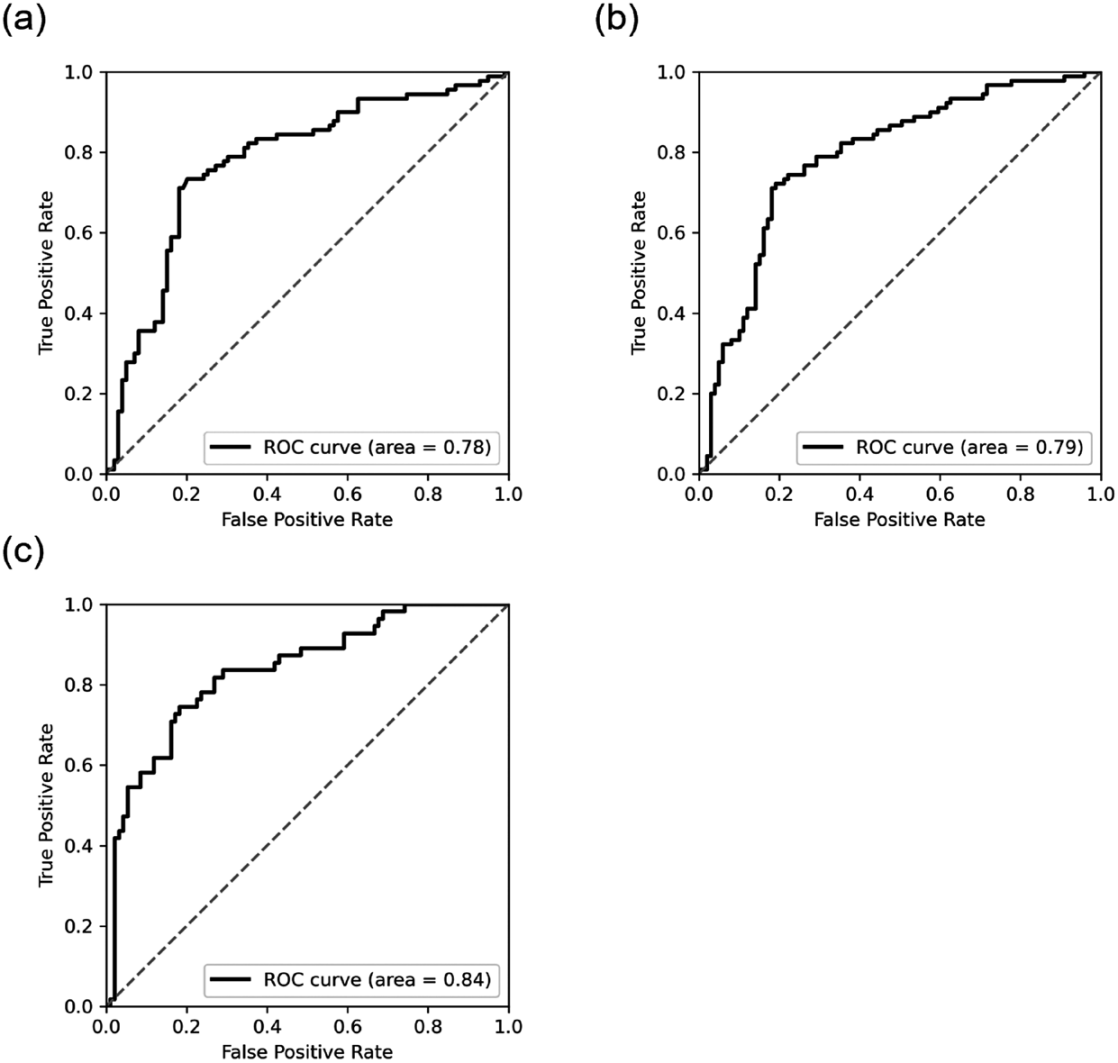
(A) The ROC curve (kernel SVM, LOOCV) when the criterion for sus-MCI was set to MMSE ≤ 26. (B) The ROC curve (kernel SVM, LOOCV) when the criterion for sus-MCI was set to MMSE ≤ 27. (C) The ROC curve (kernel SVM, LOOCV) when the borderline MMSE scores of 26 and 27 were excluded. LOOCV, leave-one-out cross-validation; MMSE, Mini-Mental State Examination; ROC, receiver operating characteristic; sus-MCI, suspected mild cognitive impairment; SVM, support vector machine.

When using MMSE to classify sus-MCI, the criteria can be ambiguous, with some studies using MMSE ≤ 26 and others using MMSE ≤ 27.^23^ Although our study adopted the former criterion, recent studies more often used the latter. Therefore, we investigated the performance when the criterion was set to MMSE ≤ 27, and the model exhibited similar performance (Figure 5: a: MMSE ≤ 26, b: MMSE ≤ 27). Additionally, because the ambiguity of the criteria makes it very difficult to classify the boundary regions of MMSE scores 26 and 27, we also evaluated the performance of the model trained by excluding participants with MMSE scores of 26 and 27 (Figure 5c). The model excluding the boundary region achieved an AUC of 0.84, demonstrating higher performance compared to the model that included the boundary region.

## DISCUSSION

In this study, to establish a high-precision MCI differentiation method using peripheral blood D-amino acid profiles, we conducted a cross-sectional study involving men and women aged 50-89 years classified as having normal cognition or sus-MCI based on MMSE scores. We explored constructing nonlinear ML algorithms using D-amino acid profiles from fingertip blood samples and noninvasive participant information. The ANN model, which demonstrated the highest performance, achieved an AUC of 0.79. Considering the performance of prior studies and the goal of utilizing this model as a prescreening tool for MCI diagnosis, it is considered valuable for real-world application. The developed model could potentially serve as a preliminary MCI screening tool before invasive and costly brain imaging and CSF Ab and tau protein measurements.

The mechanism of action of D-amino acids on cognitive function is not clear, and further research is needed. Nevertheless, some results from our cross-sectional study support previous findings on the relationship between D-amino acids and cognitive function.^16,24^ The results tested by the Mann-Whitney U test were partially different from those by the ANCOVA, suggesting an interaction between D-amino acids (%) and participant background information such as age, years of education, and number of people living together as covariates. Although there is previous research that D-glutamic acid is associated with cognitive function,^25^ our study was under the limit of detection, therefore we analyzed Ser, Ala, and Pro. In this study, D-glutamic acid was not detected in peripheral blood, which is inconsistent with previous reports; however, the reason may be due to the difference in the measurement system used in our study which was liquid chromatography-mass spectrometry, and we will further investigate this point in the future. Additionally, by investigating men and peripheral blood, for which there is no previous data, our findings suggest the utility of D-amino acids as a cognitive function assessment marker.

When comparing D-amino acid (%) in peripheral blood and venous blood, the correlation coefficient for D-Ser (%) was low. Some amino acids have different concentrations in plasma and blood cells.^26^ Furthermore, Sato et al. reported that among amino acids, the concentrations of aspartic acid, glutamic acid, ornithine, Ser, and taurine differed significantly between venous plasma and peripheral plasma. This might be because peripheral blood takes longer to collect, leading to decomposition by endogenous enzymes. Contamination from L-Ser in natural moisturizing factors present in the skin may also influence D-Ser (%). Since D-Ser has been reported to be involved in N-methyl-D-aspartate (NMDA) receptor function,^27,28^ we focused on the relationship between D-Ser and cognitive function, but in this study, there were no group differences in D-Ser enantiomeric ratios between sus-MCI and CN subjects.

In this study, nonlinear models such as RF, kernel SVM, and ANN all demonstrated higher performance compared to linear models. While significant differences in D-amino acid (%) were observed between the CN and sus-MCI groups, the overlap was substantial, making linear separation challenging. Among the nonlinear ML models evaluated (RF, kernel SVM, ANN), the ANN model exhibited the highest predictive performance. Random forest is effective for independent tabular data but struggles to learn complex inter-variable relationships.^29^ The data in this study involve complex interrelations between background information and enantiomer ratios. By setting a high-dimensional intermediate layer, the model captured these relationships effectively, leading to high performance.

The ANN model was constructed to separate linearly inseparable data by setting the intermediate layer to a higher dimension than the input features. A similar ML method for classification is the SVM with kernel, which maps input vectors to a higher-dimensional feature space and can be seen as a special form of ANN.^21^ In this study, the SVM with kernel outperformed the RF model in AUC. Thus, the difficult-to-linearly-separate data of background information and D-amino acid (%) enabled discrimination between sus-MCI and healthy individuals because of the higher dimensionality of the features.

An intriguing result of this study was the substantial improvement in performance when combining objectively collected participant background information compared to models using individual components. Previous research revealed sex differences in dementia onset rates,^30^ the relationship between age and cognitive decline,^31^ years of education, and cognitive maintenance,^32^ cohabitation and dementia onset,^33^ and a relationship between BMI and cognitive function.^34^

Considering recent MCI criteria,^23^ a post hoc analysis was conducted to examine model performance when setting sus-MCI to MMSE ≤ 27. The model with the new criteria showed equivalent performance, demonstrating the robustness of the sus-MCI criteria. As the boundary between CN and sus-MCI is challenging to determine with the MMSE, resulting in label accuracy issues, we also examined model performance by excluding participants with MMSE scores of 26 and 27. The model without boundary data exhibited higher performance, suggesting that the boundary region is difficult to distinguish even with our model. Accurate labeling of boundary regions is expected to enhance future performance.

In this study, using peripheral blood D-amino acid profiles and participant information from men and women aged 50-89 years, classified by MMSE as healthy or with sus-MCI, enabled the construction of nonlinear ML algorithms, indicating the potential for a high-precision, noninvasive MCI screening tool.

### Limitations

A limitation of this study is that the determination of MCI relied solely on the MMSE score. Consequently, the ML model developed in this study does not distinguish MCI based on a clinical diagnosis by physicians. Future research should focus on conducting cross-sectional studies involving individuals clinically diagnosed with MCI to develop a model that can accurately differentiate MCI. Additionally, there is a need to consider how to handle instances where data used in the model, such as years of education, may be missing during actual clinical diagnosis.

## Supplementary Material

pyaf016_suppl_Supplementary_Figure

## Data Availability

The data are not publicly available due to ethical restrictions. Because of the nature of this research, participants of this study did not agree to have their data shared publicly, and thus supporting data are not available.
