# Supplementary material for: Examination of the relationship between D-amino acid profiles and cognitive function in individuals with mild cognitive impairment: a machine learning approach
Source: Int J Neuropsychopharmacol. 2025 Mar 15;28(4):pyaf016. doi: 10.1093/ijnp/pyaf016 (PMC12012366; doi:10.1093/ijnp/pyaf016)
Supplement: pyaf016_suppl_Supplementary_Figure [file pyaf016_suppl_supplementary_figure.zip › revised_Supplementary_figure/revised_Supplementary_figure.pptx]

## Slide 1
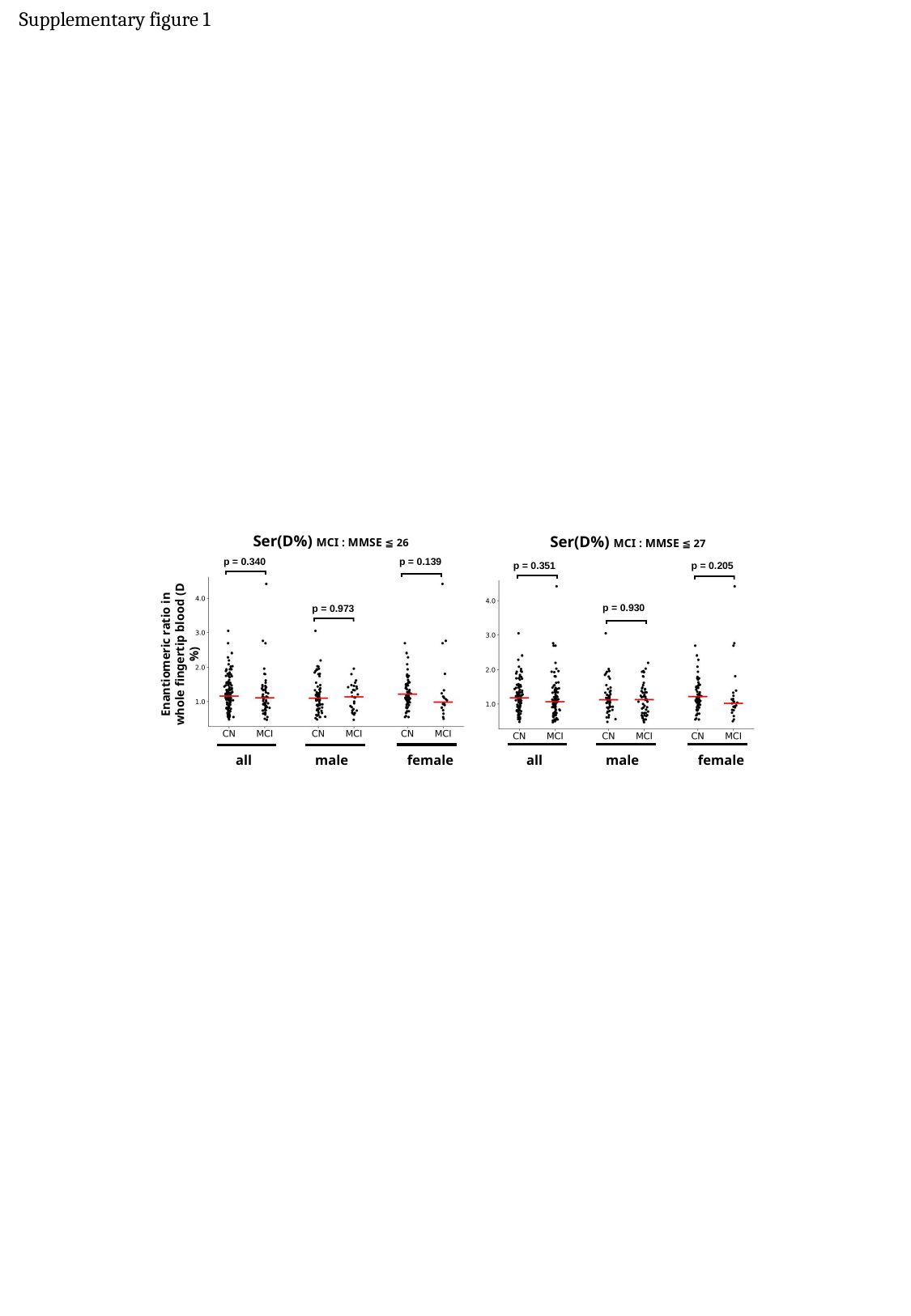

Supplementary figure 1
Ser(D%) MCI : MMSE ≦ 26
Ser(D%) MCI : MMSE ≦ 27
p = 0.340
p = 0.139
p = 0.205
p = 0.351
p = 0.930
p = 0.973
Enantiomeric ratio in whole fingertip blood (D%)
all
male
female
all
male
female
