# Supplementary material for: Examination of the relationship between D-amino acid profiles and cognitive function in individuals with mild cognitive impairment: a machine learning approach
Source: Int J Neuropsychopharmacol. 2025 Mar 15;28(4):pyaf016. doi: 10.1093/ijnp/pyaf016 (PMC12012366; doi:10.1093/ijnp/pyaf016)
Supplement: pyaf016_suppl_Supplementary_Figure [file pyaf016_suppl_supplementary_figure.zip › revised_Supplementary_figure/Supplementary figure 1.docx]

Supplementary figure 1

Scatterplots showing group differences in the concentration of D-Ser (％) in whole fingertip blood. Black horizontal lines denote the median value. Differences in each concentration between the cognitively normal (CN) group and the suspected-MCI group (MCI) by sex were explored using the Mann–Whitney U test.
